# Self-renewal and differentiation in squamous cell carcinomas

**DOI:** 10.18632/aging.102381

**Published:** 2019-10-18

**Authors:** Ana Sastre-Perona, Steven Hoang-Phou, Markus Schober

**Affiliations:** 1 The Ronald O. Perelman Department of Dermatology, NYU School of Medicine, New York, NY 10016, USA; 2 Department of Cell Biology, NYU School of Medicine, New York, NY 10016, USA; 3 Perlmutter Cancer Center, NYU School of Medicine, New York, NY 10016, USA

**Keywords:** PITX1, SOX2, KLF4, cancer stem cells, transcriptional networks, self-renewal, squamous cell carcinoma

Cancer is a heterogeneous disease comprised of a variety of cell types. It is maintained by stem cell-like tumor propagating cells (TPCs), which self-renew to sustain long-term tumor growth and differentiate into tumor cells with limited proliferative potential. How these cancerous, growth-driving TPCs are specified and how their increased self-renewal and aberrant differentiation programs are established and maintained remains elusive for most human cancers. We recently discovered a squamous cell carcinoma specific PITX1-SOX2 and KLF4 dependent, bi-stable transcriptional network, which sheds new light onto the transcriptional circuits that increase self-renewal, inhibit differentiation, and thereby allow for clonal expansion and unlimited cancerous growth in mouse and human squamous cell carcinomas (SCCs) [1].

**Figure 1:**
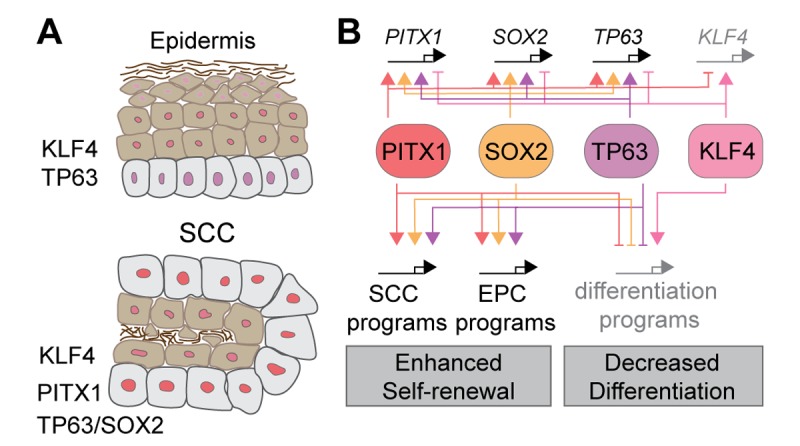
**Bi-stable self-renewal and differentiation networks define SCC growth.** (**A**) Stratified epithelia like epidermis express TP63 in proliferative basal, and KLF4 in post-mitotic, supra-basal cell layers. SCC formation coincides with de novo *Pitx1* and *Sox2* expression. PITX1 and SOX2 co-localize with TP63 in nuclei of basal TPCs, until their expression fades and KLF4 strengthens allowing TPCs to differentiate into SCC cells without proliferative potential. (**B**) The fate choice between TPC self-renewal and differentiation is governed by a bi-stable transcriptional network where PITX1, SOX2, and TP63 enhance each other’s transcription to drive self-renewal as they repress and are repressed by a KLF4 dependent squamous differentiation program.

Skin epithelial cells are the most common site of malignant transformation in humans and they emerged as a paradigm in which tumor initiation, maintenance, and progression can be determined. The malignant transformation of normal skin epithelial stem and progenitor cells (EPCs) into SCC cells involves the acquisition of oncogenic mutations, which hyper-activate the RAS-MAPK pathway, along with additional tumor suppressive mutations and stereotypic transcriptional changes. Once SCCs form, they are maintained by basal TPCs, which proliferate to self-renew, or differentiate and stratify into supra-basal SCC cells without proliferative potential (Figure 1A).

This fate choice between stem cell renewal or differentiation defines growth in these tumors and is governed by sequence specific transcription factors that co-operatively bind to lineage defining gene regulatory elements to control the expression of cell type specific transcriptional programs. Direct comparisons between TPCs and EPCs unveiled stereotypic differences in chromatin accessibility [1–3] and active gene regulatory enhancers [4,5]. These differences in gene regulation are also reflected in the expression of distinctive gene expression signatures, which molecularly and functionally distinguish TPCs from EPCs [6]. Contained within the TPC signature we discovered the transcription factors *Sox2* and *Pitx1*, which become de-novo expressed in TPCs, while they are epigenetically repressed and undetectable in normal skin epithelial cells [7].

We identified PITX1 [1] along with SOX2 [7,8] as functional TPC markers in mouse and human SCCs. PITX1 and SOX2 are specifically detected in nuclei of TPCs and their expression fades once TPCs change their fate and differentiate into post-mitotic SCC cells (Figure 1A). PITX1 and SOX2 functions are essential for SCC initiation, TPC self-renewal and SCC growth, as their genetic inhibition leads to cell cycle withdrawal, apoptosis and squamous differentiation.

This raises the question how PITX1 and SOX2 promote SCC initiation and growth? Our biochemical data suggest PITX1 can physically interact with SOX2 and TP63, a master regulatory transcription factor that specifies the stratified epithelial lineage in normal skin and its tumors. Consistent with this finding chromatin immuno-precipitation followed by sequencing (ChIP-seq) identified PITX1, SOX2 and TP63 bound at SCC specific gene regulatory enhancers where they control the expression of SCC signature genes known to enhance cell proliferation and survival, while they repress squamous differentiation (Figure 1B). These studies also revealed that PITX1-SOX2-TP63 can co-operatively bind to their own gene regulatory enhancers to support their own transcription as part of an auto-regulatory feed-forward circuit that maintains stemness and self-renewal in SCCs [1]. In addition, PITX1 function inhibits TPC differentiation, as it binds cooperatively with SOX2 and TRP63 to *Klf4* enhancers to suppress its transcription and KLF4 dependent squamous differentiation. Consistent with this model, genetic *Pitx1* inhibition interrupts the PITX1-SOX2-TP63 feed forward circuit, diminishes SOX2 and TP63 expression, and permits increased KLF4 expression, cell cycle withdrawal and squamous differentiation.

Although these findings shed new light onto the mechanisms that increase self-renewal, the root cause of aberrant SCC growth, several new questions emerge. One of these questions is how the SCC defining epigenome along with the self-renewal and differentiation defining bi-stable transcriptional network are established? One possibility is that oncogenic mutations drive the expression of pioneer transcription factors that systematically reprogram the normal into a tumorigenic epigenome to eventually express *Pitx1* and *Sox2* to drive self-renewal. Alternatively, spurious changes in gene transcription might accidentally lead to the expression of PITX1 and SOX2, which stabilize each other’s transcription, thereby selecting forever expanding TPC clones. Indeed, the low efficiency at which SCCs form from oncogenic Ras expressing clones supports the stochastic selection model. Still further studies will be required to determine how the TPC defining transcriptome is established.

A separate question with tremendous medical potential is how some TPCs remain able to break the PITX1-SOX2 self-renewal circuit to differentiate into post-mitotic SCC cells. Identification of the tumor suppressive mechanisms that limit SOX2 and PITX1 expression and function to basal SCC layers might allow the development of pharmacological approaches that can limit TPC self-renewal as they enforce their differentiation into SCC cells without proliferative potential. Answers to these emerging questions will provide new conceptual insights into the development of novel, TPC differentiation-based therapies for some of the most common and often deadly human cancers.
